# EFFORTS TO INCREASE GERONTOLOGY DEGREE-SEEKERS AND DEVELOP THE LOCAL WORKFORCE

**DOI:** 10.1093/geroni/igae098.3109

**Published:** 2024-12-31

**Authors:** Lisa Hall

**Affiliations:** Missouri State University, Springfield, Missouri, United States

## Abstract

Since 1990, Gerontology majors at Missouri State University (MSU) have served their practicums at a variety of sites, which we consider to be “community partners”. These community partners consist of public and private entities that serve older adults, ranging from the local Area Agency on Aging, which is authorized by the Older Americans Act, to caregiving individuals who own Limited Liability Companies (LLC). The services offered range from high-activity recreation to end-of-life care. The MSU Gerontology Program has routinely surveyed our community partners to understand their expectations for and opinions about our program and students. In 2023, we added two additional areas of inquiry: (1) their current networking practices with each other and (2) the degree to which they and their employees were interested in taking aging-related courses at our university. Results from our online survey and follow-up interviews will be shared. Next steps in our continued efforts to increase Gerontology degree-seekers and to develop the local age-related workforce will be discussed.

